# Guest Editorial title: UK Welfare Reform and Mental Health: A Growing Public Health Concern

**DOI:** 10.1192/bjp.2026.10698

**Published:** 2026-06-18

**Authors:** Dionne Laporte, Sarah Ledden, Matt Sutton, Síofra Peeren, Sumaty Hernandez Farina, Jayati Das-Munshi

**Affiliations:** Department of Psychological Medicine, Institute of Psychiatry, Psychology and Neuroscience, King’s College London, UK; Population Mental Health Consortium, Population Health Improvement UK (PHIUK); Department of Psychological Medicine, Institute of Psychiatry, Psychology and Neuroscience, King’s College London, UK; Population Mental Health Consortium, Population Health Improvement UK (PHIUK); Population Mental Health Consortium, Population Health Improvement UK (PHIUK); University of Manchester; Population Mental Health Consortium, Population Health Improvement UK (PHIUK); Traumascapes; Health Services and Population Research Department, Institute of Psychiatry, Psychology and Neuroscience, King’s College London, UK; Population Mental Health Consortium, Population Health Improvement UK (PHIUK); Thrive LDN; Department of Psychological Medicine, Institute of Psychiatry, Psychology and Neuroscience, King’s College London, UK; Population Mental Health Consortium, Population Health Improvement UK (PHIUK); ESRC KCL Centre for Society and Mental Health, UK

Michael O’Sullivan^1^, Kevin Gale^2^, and Nazerine Anderson^3^ may not be widely recognised names, but their stories share a tragic commonality: each died by suicide. Inquiries into these deaths highlight longstanding mental health issues and identified difficulties in navigating the UK welfare system as a contributing factor in their deaths. Their deaths illustrate the human cost of policy decisions that prioritise austerity and conditionality over social support. They also reflect a broader pattern in which welfare reforms have instead intensified distress, financial insecurity, and despair.

As the government reviews proposals to reform disability benefits, these concerns are becoming urgent. Although eligibility for disability benefits is not related to employment, pathways to work are core to the discussion surrounding intended reforms. Amid rising economic inactivity, partially due to increasing rates of mental ill-health, it is essential to critically examine the potential impact of these reforms.

This article draws on our experience across mental health research, research on work, welfare and health, clinical practice, and policy. Our perspectives are shaped by engagement with people affected by welfare reforms and ongoing work on the social determinants of mental health. We approach this discussion from a standpoint prioritising equity, inclusion, and the meaningful involvement of lived experience in evidence, policy, and clinical service delivery.

## Is Mental Health at Risk under New Welfare Reform Proposals?

Across many high-income countries, mental ill-health has become a leading driver of health-related economic inactivity^4^. The UK reflects this wider trend, with depression and anxiety now the most reported health conditions in the working-age population but since COVID-19, economic inactivity due to ill health or disability has remained elevated in the UK while stabilising or falling in countries such as Germany and France^4^. Against this backdrop, the UK government initially planned to reduce disability benefit spending by £5 billion by 2030, claiming that proposed reforms would make the welfare system more sustainable, curb rising costs, reduce economic inactivity and encourage people with health conditions and disabilities back into work. Public and political opposition to the proposals, however, led to the Government U-turn on these plans.

Welfare reform is never a purely fiscal process - it reflects political and moral ideas about work, responsibility, and who is considered “deserving” of state support. Across many high-income countries, public debates around disability and incapacity benefits have increasingly drawn on narratives that distinguish between the “deserving” and “undeserving” poor. These narratives frame reduced welfare spending not only as an economic necessity, but as a moral imperative, particularly where claimants are perceived as capable of work.

In several high-income countries, disability and incapacity benefits have been restructured around stricter assessments and behavioural conditionality. UK welfare policies also reflect this trend - with previous reforms involving stricter eligibility criteria and increased conditionality for accessing disability and incapacity benefits. Although disability benefit is not means-tested nor subject to work-related conditionality, many recipients report that the disability benefit assessment process is highly stressful, citing difficulties such as confusing assessment questions and unhelpful interactions with assessors. For many, the repeated need to demonstrate one’s “deservingness” can exacerbate mental health problems.

Evidence of the benefits of these reforms is contested. An international review of disability benefit reforms across several OECD countries found limited evidence that tightening eligibility or conditionality increased employment among people with chronic illnesses and disabilities. UK studies have indicated that benefit receipt was associated with rising food insecurity and worsening mental health, while finding limited impact on employment rates. However, other analyses suggest a more nuanced picture: single parents receiving benefits were more likely to be in work within six months of making a claim compared to those on legacy benefits, with a stronger employment effect observed for single parents than for single adults without children. These inconsistent findings highlight the uneven impact of benefit reforms, with some groups experiencing employment gains and others increased financial strain and poorer mental health outcomes.

The inconsistency in the evidence raises questions about the effectiveness of welfare reform as the primary means for addressing economic inactivity. The Mayfield Review suggests that reducing economic inactivity requires attention to workplace conditions, emphasising early intervention, inclusive workplace practices, and support for people with long-term conditions. Its focus on employer responsibility suggests that reducing economic inactivity may require reform of the broader employment system. However, this approach is likely to have limited impact for those who are already economically inactive due to long-term illness or disability, for whom re-entry into work may be shaped by entrenched health, skills, and labour market barriers.

In contrast, qualitative research showed that people’s employment barriers rarely arise from mental ill-health alone^5^. Instead, participants described a combination of challenges that include insecure housing, trauma, discrimination and transport difficulties. These factors often interact with mental distress, impacting a person’s capacity to work. When welfare systems treat ill-health as the only acceptable reason for not working, people feel pressured to present their struggles as medical issues, even when the real barriers are social or economic. This reinforces the medicalisation of socioeconomic hardship, restricts access to appropriate support, and increases distress^5^.

## Evidence from past reforms: Lessons ignored

### Benefit Eligibility

Across high-income countries, systems used to assess eligibility for disability and incapacity benefits have been criticized for prioritising physical health and often neglecting the nature and impact of mental ill-health. Research shows that individuals with mental health diagnoses are 2.4 times more likely to lose disability benefits when transitioning from legacy to newer benefit systems than those with physical health conditions^6^. One study indicated that there were six additional suicides, 2,700 more reported cases of mental ill-health, and over 7,000 additional prescriptions for antidepressants for every 10,000 individuals reassessed for their capability to work^7^. If welfare practices fail to adequately capture mental health difficulties or become more stringent, reforms risk worsening mental health and deepening socioeconomic inequalities.

### Welfare Conditionality and Sanctions

Welfare conditionality which refers to the requirements placed on benefit recipients to undertake specific activities, such as attending appointments or applying for jobs, to receive financial support. Conditionality in the UK was ranked the second strictest among OECD countries, with benefit recipients facing stricter requirements to search for and accept work. Although research on conditionality and mental health is limited, existing evidence raises concerns. One study found that imposing sanctions-backed work requirements on lone mothers with school-age children was associated with adverse effects on mental health. Further evidence suggests that conditionality is largely ineffective at moving individuals with mental health conditions into paid employment and may worsen mental health^8^.

Failure to meet these conditions may result in sanctions that penalise non-compliance. These sanctions have been criticised for their disproportionate nature - for instance, suspending payments for four weeks due to a missed appointment with an employment advisor. Between 2018 and 2019, 2.4% of benefit recipients were sanctioned, compared to 0.03% of recipients on legacy incapacity benefits and further analysis have indicated regional and ethnic differences in sanctions rates across the UK. Other research has indicated that area-level increases in sanction rates in October 2012 were associated with elevated levels of anxiety and depression. While more research is needed, conditionality and sanctions may worsen mental health outcomes and socioeconomic disadvantage.

Active Labour Market Policies (ALMP) are another strategy used by governments typically made up of supportive measures such as wage subsidies and employment support programmes like the UK Work and Health Programme. However, research suggests that the effects of ALMPs are variable. In Denmark, ALMP improved earnings among newly unemployed but had limited impact among those who were long-term unemployed and those experiencing mental health problems. In Germany, individuals with severe mental illness showed the greatest gains in employment and income from ALMP participation. UK research indicated that ALMP did not confer the same mental health benefits for men in stable, high-quality employment, whereas women experienced mental health improvements comparable to regular work. Although ALMPs are not expected to improve mental health, participation should not lead to deterioration, particularly among those already experiencing mental health problems.

## Welfare Reforms - Cost Cutting or Cost Shifting?

Welfare reforms have overlooked consequences for the healthcare system. Individuals already facing socioeconomic disadvantages - such as low income, insecure employment, inadequate housing - are at increased risk of experiencing further financial hardship following welfare reform. The financial strain can exacerbate mental health problems and increase demand for healthcare services.

International evidence indicates that tightening eligibility to disability benefits was associated with modest increases in employment. However, many people who lose access to disability benefits do not move into sustained work and are often shifted onto other, typically less generous, forms of support, or remain out of work altogether. This can increase financial insecurity, particularly among those with existing health conditions or limited labour market opportunities.

UK mental health services have struggled with persistent underfunding, exacerbated by austerity policies, and service delivery models that often fall short of meeting the complex needs of those accessing care. Welfare reforms that reduce income security may therefore place additional pressure on an already stretched healthcare system. An analysis by the Disability Policy Centre suggests that cuts to disability benefits could lead to an estimated £1.2 billion in increased expenditure for NHS and social care services. Cutting social support may reduce short-term spending but risks worsening health inequalities, intensifying pressure on under-resourced services, and shifting rather than reducing costs.

## Towards Welfare Models that Promote Mental Health

The current trajectory of welfare reform - centred on conditionality, sanctions, and increasingly restrictive eligibility - risks increasing mental ill-health, poverty and widening health inequalities. Welfare systems should be designed around financial security, dignity, upstream determinants of health (e.g. housing, healthcare access) and enabling support that promotes favourable health outcomes. Emerging evidence suggests that Universal Basic Income (UBI) - a system of regular, unconditional cash payments to residents - has the potential to confer substantial health benefits at a population level^9^. Qualitative research indicates that individuals from low-income communities anticipate material security under UBI would reduce stress, improve health, and limit shame associated with the current UK welfare model. Other systems could include expanded access to supported employment programmes which provide person-centred assistance to help people with mental health conditions engage with work. Supported employment aligns closely with several government initiatives. Increasing access to psychological support services that many participants require, while improvements to occupational health provision help employers sustain people in work through appropriate adjustments. Such approaches should aim not to compel individuals into work at any cost, but to create the social and psychological conditions in which economic participation becomes possible and meaningful.

## Conclusion

With a spotlight on welfare reforms, it is crucial to reflect on the legacy of past policies to inform more effective and equitable future welfare policies. The cases of Michael O’Sullivan, Kevin Gale, and Nazerine Anderson show how welfare processes can contribute to financial insecurity, undermine dignity, and harm health. With mental ill-health now a leading cause of long-term sickness and economic inactivity, the implications of welfare policy for public mental health can no longer be treated as secondary concerns.

Empirical evidence alone cannot determine the future of welfare provision. Decisions about social support are inevitably shaped by values, ideology, and political narratives surrounding work, responsibility, and deservingness. Empirical findings can inform policy, but they cannot resolve ongoing debates about the role of the state in social support or who is “deserving” of this support. A more effective and humane welfare system would recognise the diverse and often interacting barriers that shape people’s capacity to work. These include not only health conditions but also challenges related to housing, childcare, transport, education, discrimination, and the design of the welfare system itself. The reforms should aim to balance economic objectives with strategies that protect mental health and promote social inclusion thereby also supporting employers to foster inclusive and healthy working environments. Doing so offers the opportunity to build a welfare system that is effective, proportionate, and responsive to the diverse needs of the communities it serves.
